# Nutraceutical Applications of *Withania somnifera*: The Scientific Knowledge for Rational Modern Use of the “Ayurvedic Adaptogen”

**DOI:** 10.3390/foods15122192

**Published:** 2026-06-17

**Authors:** Sudip Pandey, Poonam Pant, Giovanni Corbioli, Erica Bonazzi, Miluska Cisneros-Yupanqui, Paola Salmaso, Stefano Dall’Acqua

**Affiliations:** 1 Wood Science Laboratory, Forest Biomaterials Science and Engineering Program, Institute of Applied Sciences, Madan Bhandari University of Science and Technology, Chitlang 44100, Nepal; sudip.pandey@mbust.edu.np; 2 Department of Pharmacy, CiST College, Kathmandu 44600, Nepal; pantpoonam91@gmail.com; 3 Solgar Italia Multinutrient S.p.A., Via Prima Strada, 23 int. 3, 35129 Padova, Italy; giovanni.corbioli@it.nestle.com (G.C.); erica.bonazzi@it.nestle.com (E.B.); miluska.cisneros@it.nestle.com (M.C.-Y.); paola.salmaso@it.nestle.com (P.S.); 4 DSF Department of Pharmaceutical and Pharmacological Science, University of Padova, 35122 Padova, Italy

**Keywords:** herbal medicine, dietary supplements, standardization, withanolides, bioavailability, stress-related outcomes, sleep quality, neuroprotection, safety assessment, regulatory framework

## Abstract

*Withania somnifera* L. (Dunal), commonly known as Ashwagandha, is widely used in traditional medical systems, particularly Ayurveda, and is increasingly included in nutraceuticals and dietary supplements. This narrative review summarizes and critically discusses the literature published from 2015 to 2026 on WS, with a focus on CNS-related outcomes, proposed mechanisms, extract standardization, dosage, and safety considerations. Evidence from preclinical research and human studies suggests that WS preparations may influence stress-related and sleep-related outcomes and support neuroprotective pathways suggesting a significant role in nutraceuticals; however, the overall strength of evidence varies across indications and products, and heterogeneity in extract composition and study design limits firm conclusions. Further well-designed, adequately powered clinical trials using standardized preparations are needed to clarify efficacy, mechanisms of action, and long-term safety.

## 1. Introduction

*Withania somnifera* (L.) Dunal has gained significant attention in recent years for its numerous applications in traditional medicine, as well as in food supplements and nutraceuticals, particularly for its energizing effects, enhancement of sports performance, and benefits to central nervous system (CNS) [1,2,3,4,5,6,7,8]. Historically, it is recognized as Rasayana, which translates to “tonic” in Ayurveda, and it is claimed for its adaptogenic properties, supporting stress management and being used for the improvement of overall well-being [4,5]. It is useful to remember that Ashwagandha, also known as winter cherry and Indian ginseng, belongs to the Solanaceae family and has a long history in Ayurvedic medicine, having been used for over 3000 years. The Ashwagandha name is related to the specific peculiarity that its root smells like horse (“Ashwa”) urine, and popular traditions suggest that this plant can provide horse-like strength when consumed [4]. It is highly valued in Ayurvedic traditions and is regarded as an adaptogen; it helps enhance strength and brain and nervous system function, and improves sexual performance and reproductive health. WS is considered able to improve the body’s defence against disease [4]. Due to these important traditional uses, WS have attracted the attention of many researchers across different disciplines. Scientific research on WS appears to be substantial, with more than 1200 records indexed in PubMed from 2016 to the time of writing this review. This suggests considerable interest in WS, not only from the scientific community but also from consumers and industries, particularly regarding its potential health-promoting effects and, consequently, possible innovations in processes such as extraction methods, as well as into trials that can confirm its clinical efficacy and safety for human use.

WS contains a large number of bioactive compounds, including phenolic, flavonoids, sterol derivatives known as withanolides, and alkaloids, which are believed to be its active constituents [9,10]. Many reviews suggest multiple biological activities, often based on preclinical evidence concerning WS. Reported effects include anti-inflammatory activity [2,11], benefits in neurological disorders [5,7,12,13,14], support for healthy aging [2,11], and improvements in fertility-related conditions [15]. Many studies and reviews have focused on the central nervous system (CNS), showing that WS can influence immunomodulation, stress management, and physical performance [1,3,5,8,11,12,16,17,18,19]. The extensive body of scientific literature describing the potential usefulness of WS, together with the well-established knowledge of its chemical constituents [1,19,20,21,22,23], supports its growing recognition as an emerging ingredient investigated for potential health-related applications.

Despite the large number of recent reviews on WS, this paper aims to provide a novel perspective on its potential applications in nutraceuticals and food products. In this review, we compile and analyze relevant studies published between 2015 and 2026, with a particular focus on WS bioactivities and recent clinical trials involving human subjects. The objective is to critically evaluate the scientific evidence supporting the claimed effects and to highlight both the strengths and limitations of this plant species, which may be beneficial in various conditions but requires proper understanding for appropriate use. PubMed and Scopus were used as primary sources for the literature search. A brief overview of the chemical constituents is also included, as this is essential for understanding the potential mechanisms of action of WS. Finally, specific conclusions are drawn regarding its efficacy, health-promoting properties, and safety in nutraceutical applications.

### Literature Search Strategy

This narrative review is based on literature searches performed in PubMed and Scopus. The core search mostly is referred to the period 2015–2026 and focused on Ashwagandha/WS in the context of nutraceutical/food supplement use and CNS-related outcomes. We prioritized human clinical trials, systematic reviews/meta-analyses, and well-characterized preclinical studies addressing mechanisms, extract standardization, dose, and safety. Studies were screened for relevance to nutraceutical applications; evidence was interpreted by separating in vitro, in vivo, and human data and by highlighting heterogeneity in extract composition and study design.

## 2. *Withania somnifera* (L.) Dunal (WS): Botanical Profile and Ethnopharmacological Use

*Withania somnifera* (L.) Dunal (WS) is a perennial herbaceous plant native to the arid and semi-arid regions of India, the Middle East, and parts of Africa. A recent review by Mukherjee summarized the Ayurvedic importance and ethnopharmacological relevance of this plant [4]. The use of WS in Ayurveda is largely reported in several texts in which formulations with various dosage forms are mentioned [4]. The literature indicates that WS has moved from its ancient use to recent applications, where it is considered a promising remedy for human health and is used to maintain health and wellness. WS-based formulations are extensively marketed in many countries of the world in different forms, mostly registered as herbal medicine or supplements formed mainly of plant extracts or powdered material [1,2,3,4,5].

It belongs to the kingdom Plantae, the sub-kingdom Tracheophytes (vascular plants), division Angiosperms, class Eudicots, clade Asterids, order Solanales, family Solanaceae, sub-family Solanoideae, tribe Physaleae, genus *Withania*, and species *somnifera* [24,25,26]. The plant typically attains a height of about 30 to 150 cm and is characterized by ovate leaves measuring up to 10 cm in length [27]. The leaves are densely pubescent on the abaxial surface and exhibit a bitter taste, with margins ranging from completely straight to slightly wavy. The roots of WS are long (15–25 cm), cylindrical, and exhibit a brownish-white to light yellow coloration, accompanied by a characteristic horse-like odor and bitter taste [28]. The roots are the most pharmacologically significant part of the plant and are widely recognized for their adaptogenic, anti-inflammatory, antioxidant, and neuroprotective properties [1,2,3,8,16,17,19,22,24,25,29]. Historically, WS has been classified as a “Rasayana” in Ayurvedic medicine, indicating its role in promoting longevity, vitality, and resistance to stress. Contemporary studies further highlight its potential in enhancing physical performance and managing stress-related disorders through its neuroprotective, antioxidant, and immunomodulatory activities. In addition to its extensive pharmacological investigation, *Withania somnifera* represents a paradigmatic example of a traditional ethnopharmacological remedy that has been progressively translated into modern nutraceutical applications, raising specific issues related to standardization, safety, and regulatory positioning.

**Figure 1:**
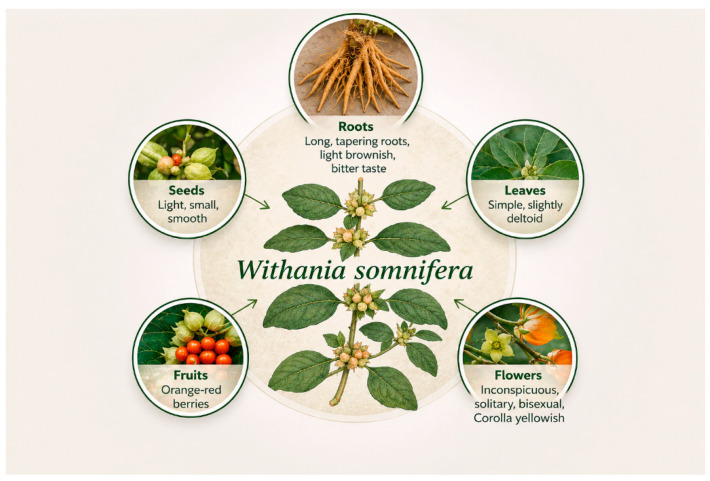
Botanical description of WS.

The Indian Ayurveda Pharmacopeia specifically defines Ashwagandha as the dried mature roots of WS, thereby standardizing its pharmacological use and distinguishing it from other plant parts, such as leaves and berries, which differ in chemical compositions and therapeutic properties [30]. The plant produces small, greenish-yellow flowers that develop into orange-red berries about the size of a raisin [31] (Figure 1). It is common in dry tropical regions including Afghanistan, Baluchistan, the Canary Islands, China, Congo, Egypt, India, Iran, Israel, Jordan, Madagascar, Morocco, Nepal, Pakistan, South and East Africa, Spain, Sri Lanka, Sudan, and Yemen [24,32].

Seeds of WS are small, compressed, reniform, yellow in color, and vary from reticulate to smooth in surface texture [33,34,35]. A recent review summarized the use of various parts of WS including roots, seeds, stem bark, and stems, in traditional medicinal preparations for the treatment of different diseases [5]. These plant materials are processed into a variety of formulations, such as decoctions, powders, and pastes, and are administered either orally or topically depending on the therapeutic purposes [5]. Notably, several traditional remedies in India involve the topical application of WS. For example, pastes prepared from leaf roots are used for the treatment of leprosy, while root powder, decoction, leaf paste, ash, and root powders mixed with milk are applied topically to alleviate weakness, pain, and certain female disorders [5]. In children, respiratory ailments are traditionally treated with root paste boiled in mustard oil or preparations containing roots, cardamom seeds, and Kumkum kesari boiled in cow butter. Other reported topical applications in India are primarily associated with the management of inflammation and pain across a range of conditions [5]. The variation in phytochemical compositions among different plant parts accounts for their distinct medicinal properties and applications. Differences in plant origin and plant part can cause variations in the chemical composition, with changes in efficacy [24]. This is an issue that is relevant for almost all the botanical ingredient, and supports the crucial need for standardized extraction and analytical procedures to establish the number of bioactive compounds in the products. There are many reviews summarizing the bioactivities of WS [1,2,3,4,5,8,9,11,24], and the most important claimed activities are adaptogenic, antistress, neuroprotective, as a treatment for male infertility, anti-inflammatory, antidiabetic, and cardiotonic [24].

## 3. Phytochemical Composition and Bioavailability of WS Extracts for Nutraceutical and Pharmaceutical Applications

Bioactivity of plant-derived preparations and herbal medicine is strictly related to the chemical composition. This concept can be clearly applied to WS considering the modern application of this plant. WS has a long history in ethnopharmacological use and is now diffused in several nutraceutical formulations. Assessing its safe and efficient use requires a detailed knowledge of the phytochemical composition, extract standardization, and information about products formulations and bioactive constituents’ bioavailability.

WS phytoconstituents are numerous and the composition of the different parts of the plant has been studied through chemical analysis. Many phytochemicals have been isolated and identified using chromatographic techniques based on different mechanisms such as size exclusion chromatography and direct and reverse phase stationary phases. Analytical and preparative chromatography have been applied as thin-layer chromatography (TLC) and high-performance and ultra-high-performance liquid chromatography (HPLC, UPLC), attempting to offer the most comprehensive analysis of the phytoconstituent that can be extracted from different plant parts (leaves, twigs, etc.) [21,36,37,38,39,40,41]. Recently, the chemical composition of the root has been studied, combining Nuclear Magnetic Resonance (NMR) and Liquid Chromatography coupled with mass spectrometry (LC-MS) approaches, with a view to comparing different extraction methods to highlight the differences in the chemical composition and biological activity of the extracts [13]. Furthermore, a recent review [9] is relevant in this regard because it summarized the composition and extraction protocols useful for food applications.

The literature shows that plant material, due to its numerous health-promoting properties, contains a vast array of minerals and phytochemicals [20,42,43]: a short summary is reported in Table 1. There are several classes of chemical constituents of *Withania* and, in particular, alkaloids (isopelletierine, anaferine), steroidal lactones (withanolides, withaferins), saponins, and withanolides with glucose at carbon 27 [17,25,44]. Among them, withanolides (steroidal lactones) have been used in an increasing number of formulations, given their promising therapeutic abilities [44]. Structurally, withanolides are ergostane-type steroids that feature a lactone ring involving the oxidized C-1 position, along with atoms C-22 and C-26. The lactone ring can be six-membered, as in withanolide A, or five-membered, as in withanolide B. These compounds are relatively specific to the genus *Withania* (Solanaceae) and are often used as marker compounds, though they can also be found in a few other species [36]. The most studied withanolides include withaferin A and withanolide D, both of which have been associated with multiple bioactivities. Recently, Bashir et al. [1] reviewed the phytochemistry and molecular targets of Ashwagandha, highlighting the importance of its various phytochemical classes. Shinde [45] proposed a comprehensive review focusing on the extraction techniques of WS phytoconstituents, their pharmacological activities, and emerging trends in food applications.

**Figure 2:**
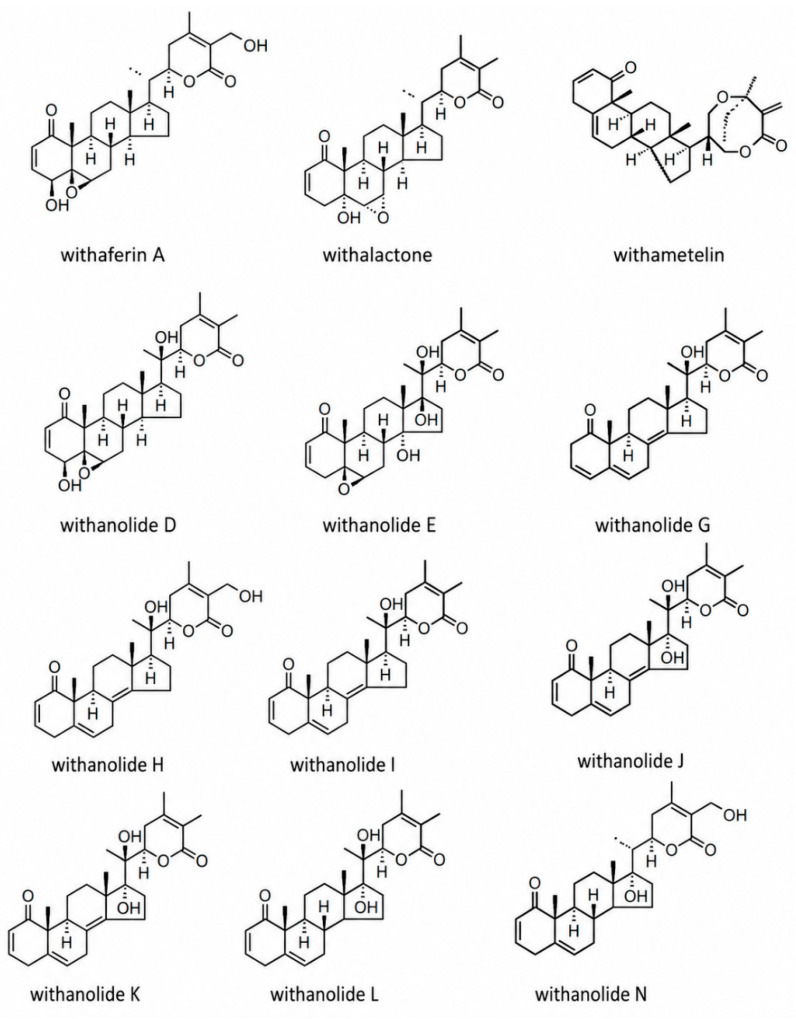
Structure of some of the most relevant withanolides.

Concerning the chemical constituents, the large number of structures that have been reported indicate the rich phytochemical composition of the plant. A significant paper reports a complete review of the withanolide chemical structures discovered and published in the time range 1965–2014; the same authors also published a precise revision of NMR data that have been published, and the papers offer a comprehensive informative review of the large number of compounds isolated and identified from this species [54,55]. Some of the most representative structures of withanolides are reported in Figure 2.

Furthermore, the previous literature reviews revealed that other compounds, such as sitoindosides, withanamides, reducing sugars, peroxidases, glycosides, starch, withanicil, benzyl alcohol, dilcitol, 2-phenyl ethanol, 3, 4, 5-trihydroxy cinnamic acid, benzoic acid, and phenyl acetic acid, are present in the plant’s extract from both its roots and leaves [20,56,57]. WS leaves are reported to contain several withanolides, condensed tannins, flavonoids, glycosides, and free amino acids [48], but the composition of leaves and roots is different, and the selection of the most appropriate vegetal material is critical to prepare safe and bioactive extracts [45]. Bessalle and Lavie [51] isolated two chlorinated withanolides, namely, withanolide C and 4-deoxyphysalolactone, from dried leaves of WS. Misra et al. [50] have found novel compounds of ergosterol and 1, 4-dioxane from WS roots, as well as different fatty acids (octacosane, oleic and stearic fatty acids), steroids, and oleanolic acid. Ethanol and methanol extracts of WS leaves and roots showed the presence of withanolide B, rosifoliol, and phytol [58]. Gulati et al. [59] studied the roots of different genotypes of WS and reported the presence of several metals in their composition, along with varying concentrations of total sugars, alkaloids, and tannins. Phytochemical investigations of ethanol and methanol extracts of WS leaves and roots showed the presence of various components, including withanolide B, rosifoliol, and phytol [60]. Fruits of WS contain amino acids, a proteolytic enzyme, condensed tannins, and flavonoids [53]. Using gas chromatography coupled with mass spectrometry (GC-MS) and NMR spectroscopy, Bhatia et al. [31] investigated the impact of chemotype changes in the chemical composition of WS fruits and reported distinct differences in the amounts of metabolites in various chemotypes. In an earlier study, Ali et al. [52] reported that the stem of the plant is rich in crude protein, calcium, and phosphorus and described, in the same organ, the presence of scopoletin.

One significant point is the selection of the starting plant material used to produce extracts, supplements, and nutraceuticals. Considering the amount of the different bioactive compounds, some authors reported that the quantity of withaferin A was 1.81 times greater in comparison to withanone in WS leaves [61]. Recently, Narayanan and Nagegowda [62] published a comprehensive review on the biosynthesis of withanolides, which provides valuable insights for biotechnological approaches aimed at producing specific withanolides. The detailed information on the biosynthetic pathway opens new possibilities for developing enriched or pure products with high levels of withanolides, which may be utilized in medicinal or functional food applications. As a simplification, roots can be considered as sources of withanolides, while leaves mostly provide withaferin derivatives. Extraction of bioactive constituents from plant material can be a challenge and can reflect on the efficacy and safety of the final products. Some patents (WO 2020/079712 A1; WO 2012/160569 Al) have been published describing the process for the extraction of highly purified extracts from WS roots [63,64]; one particular process (US006153198A) claimed high final levels of withanolide glycosides and sitoindosides, (3–8%), 3% of oligosaccharides with a molecular weight of less than 2000 Da and less than 0.5% of free cytotoxic withaferin A [65].

Various types of extracts are available on the market, and in a recent paper, Kumar et al. [66] investigated the composition and safety profile of different Ashwagandha preparations. The literature indicates that standardized WS extracts are generally recognized as safe (GRAS). However, moderate to severe toxic manifestations may occur at high dosages of extracts containing elevated levels of withaferin A. The authors recommend the use of whole-plant extracts, as formulations based on the whole plant contain additional metabolites that may help mitigate the toxicity associated with the root [66]. To date, preparations using both aerial parts and root extracts, as well as whole-plant extracts, are available. The complex phytochemical composition of this species and the high pharmacological value of the compounds suggest the critical role of the quality of the plant material, the importance of the selected plant part (roots, leaves, fruits, etc.), as well as the extraction procedures needed for purification and concentration of the phytochemicals. A recent review underlines the crucial role of the choice of WS plant part and extraction procedures in establishing WS as a valuable nutraceutical [45].

Considering one of the most studied compounds, withaferin A, it should be noted that leaves are regarded as the primary source of this steroidal lactone. Pharmacological studies, mostly in vitro and using animals, have identified withaferin A as a potent anticancer and anti-inflammatory agent, and a recent review [67] summarizes its potential role as a novel anticancer compound, particularly in gastrointestinal malignancies. The same review also highlights a critical concern regarding withaferin A, namely, its potential hepatotoxicity. Some reports have described cases of acute liver failure in susceptible individuals, underscoring the need for caution when using preparations containing high levels of this compound [68,69]. Contributing factors to hepatotoxicity may include high or non-standardized dosing, variability in phytochemical composition, and possible adulteration or contamination. Additional confounding factors include pre-existing liver disease and potential interactions with concomitant medications. Moreover, the high first-pass hepatic exposure of withanolide A and its preferential distribution to the liver may further influence an individual’s susceptibility [24,67]. Leaves contain higher levels of withanolide A and withanone and can therefore be used to prepare extracts. They are often considered an alternative to roots, as they represent a renewable source that can be harvested without destroying the plant. In addition, their distinct phytochemical composition compared to roots may yield extracts with different biological properties. The use of leaves may also reduce the risk of soil-derived contaminants, and they allow for easier identification of healthy versus diseased plant materials [70]. On the other hand, the roots represent the most traditional plant material used for WS preparations. They are also the exclusive source of withanosides (I–VII), which are considered particularly important for neuroprotective effects and the claimed memory-enhancing properties of *Withania* remedies. Most clinical trials published to date have been conducted using root extracts [1].

According to the available literature, many parts of the plant contain significant amounts of bioactive compounds. However, the roots have been predominantly used in Unani and Ayurvedic systems of medicine for a long time, reflecting a strong tradition of therapeutic application [30]. The Ayurvedic Pharmacopoeia of India (API) is an authoritative reference that sets the standards for the quality, purity, and potency of selected Ayurvedic drugs manufactured and distributed by licensed producers across India. It plays a crucial role in ensuring the standardized and regulated use of medicinal plants like WS in traditional healthcare practices [71,72]. API defines Ashwagandha as the dried mature roots of WS [72]. This confirms that, based on traditional use, the roots are the preferred part for making WS extracts. As a general consideration, the selection of plant parts is crucial. Based on API and traditional Indian preparation, the roots are the preferred plant materials. Moreover, it is strongly recommended that standardized WS extracts be used to ensure consistent levels of bioactive compounds.

The bioactive compounds present in WS are numerous, such as phenolics, withanolides, and alkaloids, and are all likely to contribute to its medicinal properties. Furthermore, large differences in the yield and concentration of extracts can be observed due to the different geographical origin, quality of plant materials, and, of course, plant organ. Recently, in a clear review, Singirala et al. [9] summarized the extraction processes of WS and reported some general values that can be useful to understand the content of plant material. In fact, the total phenolic and flavonoid content in WS root extract is around 30 mg/g of the total phenolic compounds and 17 mg/g of flavonoids. WS leaves are reported to contain around 5 mg/g for both classes of compounds. Considering all compounds, withanolide amounts ranged from 0.07 to 0.04% in roots, while values of 0.05% and 0.24% are reported in stems and leaves, respectively. Alkaloid content in Indian WS roots ranges from 0.13% to 0.31% [9].

Traditional methods for medicinal plant bioactive compound extraction include maceration, digestion, decoction, infusion, and, for limited samples, Soxhlet extraction. These are all based on solvent penetration into plant tissue, dissolution of phytochemicals, and diffusion in the solvent. More recent extraction methods have been developed to increase extraction yields and to reduce energy and time consumption and such approaches are generally all solvent-based. The most relevant methods are microwave-assisted extraction (MAE) and ultrasound-assisted extraction (UAE). A different but, in some cases, valuable approach is the one using carbon dioxide (CO_2_) in specific pressure and temperature conditions, generating supercritical fluid. This technique is called supercritical fluid extraction and allows for the extraction of lipophilic constituents with a sustainable and green approach [9]. Supercritical CO_2_ has been applied to extract fatty acid from WS roots [73]. For the preparation of WS extracts used in food supplement and nutraceutical applications, the preferred solvents are generally water or mixtures water ethanol. These solvents are GRAS and food grade.

The efficacy of herbal remedies, food supplements, and nutraceuticals is closely related to the absorption of bioactive compounds from Ashwagandha extracts. Therefore, pharmacokinetics should be considered to determine the bioavailability of the various claimed bioactive phytochemicals. Singh et al. [74] demonstrated that withanolide A is a highly permeable compound with a strong capacity to bind to plasma proteins in rats. The study also examined red blood cell uptake, showing a rapid equilibration between cells and plasma. After oral administration, withanolide A exhibits very low plasma concentration and poor bioavailability. In contrast, intravenous administration results in rapid distribution across tissues, with evidence that withanolide A can cross the blood–brain barrier in rats [74]. More recently, Modi et al. [75] proposed a validated method for simultaneously evaluating seven constituents of WS extracts in rat plasma. The pharmacokinetic and ex vivo permeability data obtained from this method are considered useful for developing clinical trials of WS in humans [75]. Kim et al. [76] explored the pharmacokinetics of WS extracts in healthy human adults following a single dose of two commercial Ashwagandha products containing equal amounts of total withanolides. They observed differences between the two extracts, likely due to metabolic bioconversion, as withanolide glycosides are converted into aglycones. The authors concluded that assessing extract composition is crucial for evaluating oral bioavailability, as shown by the pharmacokinetic profiles of the two products, and indicated that high levels of withanolide glycosides may be beneficial [76]. More recently, Vaidya et al. [6] introduced a method to assess the pharmacokinetics of withanoside IV, withanoside V, withanolide A, withaferin A, and 12-deoxy-withastramonolide following the oral administration to adult healthy volunteers of 500 mg of Ashwagandha extract containing 7.5 mg of total withanolides. This method proved effective for pharmacokinetic evaluation [6]. Overall, while progress has been made in understanding the bioavailability of withanolides, further studies are needed to clarify the bioavailability of different preparations and products. WS exhibits a complex, multi-target pharmacological profile, largely driven by its rich content of withanolides (such as withaferin A and withanolide A), alkaloids, sitoindosides, and flavonoids [1,20,77]. These bioactive compounds contribute to its adaptogenic, antioxidant, immunomodulatory, and neuroprotective properties [16].

## 4. In Vitro Studies on WS: Mechanisms and Bioactivities

In vitro studies have played a crucial role in elucidating the pharmacological and nutraceutical potential of WS, particularly by clarifying its cellular and molecular mechanisms of action. The plant exhibits a complex, multi-target pharmacological profile, largely attributed to its content of bioactive compounds such as withanolides (e.g., withaferin A and withanolide A), alkaloids, sitoindosides, and flavonoids [1,16,37,77]. These constituents are associated with adaptogenic, antioxidant, immunomodulatory, and neuroprotective properties. In vitro approaches commonly employ cell culture models, root extracts, and hairy root systems to isolate active compounds, particularly withanolide, and to evaluate their biological activities under controlled experimental conditions [78].

According to a study, the Nrf2 pathway is activated by whitanolides through intracellular signaling, leading to an increase in cellular defense antioxidants proteins, including heme oxygenase-1 (HO-1), catalase, superoxide dismutase (SOD), and glutathione peroxidase [79,80]. This redox-related response may contribute to reduced oxidative stress in cellular models, a common contributing factor to cellular aging and neurodegeneration [81]. Likewise, WS has shown anti-inflammatory and immunomodulatory activity in preclinical models. In microglial cells like BV-2, its withanolides, especially withaferin A, suppress production of ROS and nitric acid by lowering NF-kB signaling while increasing the Nrf2 response [80]. Some studies suggest that Ashwagandha supplementation may modulate immune-related markers, including macrophage activation/phagocytic activity, T-cell proliferation, and cytokine profiles; however, the magnitude and clinical relevance of these effects remain to be clarified [16,82]. Preclinical studies suggest neuroprotective effects, potentially supporting neuronal function through multiple mechanisms. In vivo and in vitro models indicate that WS extracts and isolated withanolides may attenuate neuroinflammation, reduce neuronal apoptosis, and enhance synaptic plasticity; these effects have been linked to neurotrophic signaling pathways (e.g., BDNF–TrkB, PI3K–Akt, PLCγ–IP3) [83]. An in vitro model was used to investigate WS effects on the expression of genes related to neural plasticity and HDAC2 expression. The study showed that the treatment successfully prevented HDAC2 overexpression, helping to restore normal synaptic plastic gene expression in SH-APP cells.

Multiple in vitro assays have reported antioxidant activity of WS root extracts. The hydroethanolic extracts showed notable DPPH and hydrogen peroxide scavenging effects, comparable to vitamin C used as a positive control. Total antioxidant capacity, assessed by the phosphomolybdenum assay, increased in a dose-dependent manner and was expressed as ascorbic acid equivalents. Similarly, the ferric-reducing antioxidant power (FRAP) assay indicated a concentration-dependent increase in reducing ability, confirming the extract’s potential to convert ferric (Fe^3+^) to ferrous (Fe^2+^) ions [84]. This redox-balancing effect helps reduce oxidative stress, a common contributing factor to cellular aging and neurodegeneration [81]. Recent in vitro investigations using adventitious root cultures further support these findings, demonstrating strong antioxidant and anti-inflammatory activities attributed to elevated phenolic and flavonoid content. The extracts show significant free radical scavenging (DPPH, ABTS) and inhibit key inflammatory enzymes, including proteinase and lipoxygenase. Phytochemical analyses identify catechin and gallic acid as major contributors to these bioactivities, highlighting the therapeutic potential of metabolite-enriched root cultures [85]. In vitro studies suggest anticancer potential of WS across multiple cancer cell lines, including PC-3, HCT-15, A-549, DU-145, IMR-32, and HepG2. Extracts derived from roots, stems, and leaves exhibit significant antiproliferative effects, with particularly strong activity observed in colon and liver cancer models. Adventitious root cultures enriched in phenolics and flavonoids further enhance cytotoxicity, highlighting the contribution of compounds such as catechin and gallic acid [85]. Additionally, root-derived fractions show potent cytotoxic effects against oral cancer cell lines (e.g., Ca9-22), comparable to standard chemotherapeutics, primarily through apoptosis induction and cell cycle arrest, with BRD3 and CDK2 identified as potential molecular targets [86]. Selective cytotoxicity has also been reported, with methanolic extracts exhibiting stronger effects in HepG2 cells compared to normal L929 cells, particularly for leaf and stem extracts, suggesting higher anticancer potential of aerial plant parts [87]. Mechanistically, withanolide glycosides, especially withagenin A diglucoside, play a central role by inducing apoptosis via both intrinsic and extrinsic pathways, as evidenced by caspase activation, increased Bax, and reduced Bcl-2 expression. Furthermore, these compounds inhibit angiogenesis through suppression of VEGFR2-mediated signaling pathways, including ERK, PI3K/Akt, and mTOR, supporting a mechanistic hypothesis relevant to angiogenesis- and proliferation-related pathways [88]. In vitro studies demonstrate that WS exerts significant anti-inflammatory effects in lung epithelial cell lines (NCI-H460, A549) and human peripheral blood mononuclear cells (PBMCs). Pre-treatment with root aqueous extracts markedly reduces lipopolysaccharide (LPS)-induced expression of pro-inflammatory cytokines, including TNF-α and IL-6, indicating effective modulation of inflammatory responses [89]. Mechanistically, these effects are associated with inhibition of the IKKβ/NF-κB signaling pathway, preventing NF-κB nuclear translocation and subsequent cytokine production [82]. Additionally, withanolides, particularly withaferin A, have been shown to suppress reactive oxygen species (ROS) and nitric oxide production in microglial cells by downregulating NF-κB signaling while enhancing Nrf2-mediated antioxidant responses [80]. Furthermore, evidence suggests that WS modulates immune function by enhancing macrophage activity and promoting T-cell proliferation, contributing to its overall immunomodulatory and anti-inflammatory potential. These claimed effects on the immune system are all highly valuable for nutraceutical applications.

WS protects neuronal function and supports brain health via multiple mechanisms. Preclinical in vivo and in vitro studies have shown that it can reduce neuroinflammation, inhibit neuronal apoptosis, and enhance synaptic plasticity. These effects are mediated through neurotrophic signaling pathways, especially BDNF–TrkB, PI3K–Akt, and PLCγ–IP3, which are fundamental to neuronal survival, growth, and maintenance [83]. An in vitro model was used to investigate WS effects on the expression of genes related to neural plasticity and HDAC2 expression. The study showed that the treatment successfully prevented HDAC2 overexpression, helping to restore normal synaptic plastic gene expression in SH-APP cells. A mechanistic study discussed mithramycin-related pathways in neurodegenerative experimental models; however, the relevance of these mechanisms to WS preparations and constituent profiles should be interpreted with caution [90]. Researchers suggest that compounds like withaferin A may interact directly with glucocorticoid receptors in the brain to influence cortisol and stress [91,92,93].

One of the constituents of WS, especially present in leaves, is withaferin A. This steroidal lactone has shown significant neuroprotective effects, demonstrating alleviation of TDP-43 pathology and improved cognitive function in a mouse model of Fronto-Temporal Lobar Degeneration (FTLD) [94]. This aligns with findings from Wongtrakul et al. [95], who reported that the plant exhibited neuroprotective effects in a Parkinson’s disease cell model, highlighting its ability to mitigate oxidative stress and inflammation, which are critical factors in neurodegeneration. Pharmacokinetic studies revealed the absorption and metabolism of withanolides, which are crucial for their efficacy in CNS applications [75]. Research indicates that Ashwagandha may play a role in modulating stress responses and improving cognitive function. However, the specific effects of Ashwagandha on cognitive abilities in individuals with autism have not been conclusively established in the literature. The study indicated the potential of Ashwagandha in mitigating sodium valproate-induced autism in a rodent model by improving altered behavior and reducing oxidative stress. Histological studies of the cerebellum further confirmed its ameliorative effect in autism, showing restoration in the number of Purkinje fibers, reduced neuronal degeneration, and decreased chromatolysis [96]. Computational approaches (e.g., network pharmacology, molecular docking) have explored potential interactions of WS constituents with autism-relevant targets (e.g., IL-6-related pathways). These findings are hypothesis-generating and require validation in appropriate preclinical models and, ultimately, clinical studies [97]. Several preclinical studies have explored the potential of WS extracts to modulate reward- and withdrawal-related pathways in models of substance exposure; however, clinical evidence in addiction is currently limited and does not allow firm conclusions [98,99]. It has been found to reduce nicotine-induced place preference and alleviate withdrawal symptoms in chronic alcohol exposure by modulating the GABAergic and serotonergic systems [100]. Ashwagandha was found to slow disease progression and improve motor performance in SOD1^G93A^ mice by inducing autophagy, which increased motor neuron numbers in the lumbar spinal cord. It also reduced misfolded superoxide dismutase (SOD1) levels and prevented phosphorylation of nuclear factor kappaB (NF-kB) phosphorylation [101].

## 5. Clinical Human Trials and Case Studies

Much research has been conducted on WS over the years. Although different plant parts have been explored, the roots have traditionally been the most widely used and remain the primary focus on medical research [102]. In Parkinson’s disease, the plant helps to protect against injury, mainly by reducing oxidative stress, increasing dopamine (dopaminergic D2 receptor) with an improvement in mitochondrial function [103,104]. A meta-analysis of five randomized controlled trials involving 400 participants found that WS significantly improved overall sleep quality compared to a placebo [105]. People with insomnia experienced greater improvements, and the benefits were stronger with higher doses (≥600 mg/day) and supplementation periods (≥8 weeks) [106,107,108,109].

Researchers suggest that compounds like withaferin A may interact directly with glucocorticoid receptors in the brain to influence cortisol and stress [91,92,93]. WS supplementation can raise testosterone in adults by regulating the hypothalamic–pituitary–adrenal (HPA) axis and by reducing inflammation and oxidative stress [110,111]. WS presents different bioactivities because of the multi-targeted mechanisms of its phytoconstituents. The bioactive compounds of WS with their combined effects may explain its adaptogenic, neuroprotective, and hormone-regulating benefits, making it a promising natural intervention for stress, neurodegenerative disorders, and overall well-being.

Clinical trials and meta-analyses suggest benefits on stress- and sleep-related outcomes, though with heterogeneity in extracts, doses, and populations, with additional putative benefits reported in male fertility and metabolic parameters [102,112,113] (Table 2). These effects are likely mediated through modulation of the hypothalamic–pituitary–adrenal axis, cortisol reduction, and antioxidant and neuroendocrine mechanisms [114,115,116]. Across published short-term clinical trials, WS was generally well tolerated, with mostly mild adverse events reported; however, long-term safety and product-specific risk (e.g., high withaferin A content, variability, and potential adulteration) require further study.

**Figure 3:**
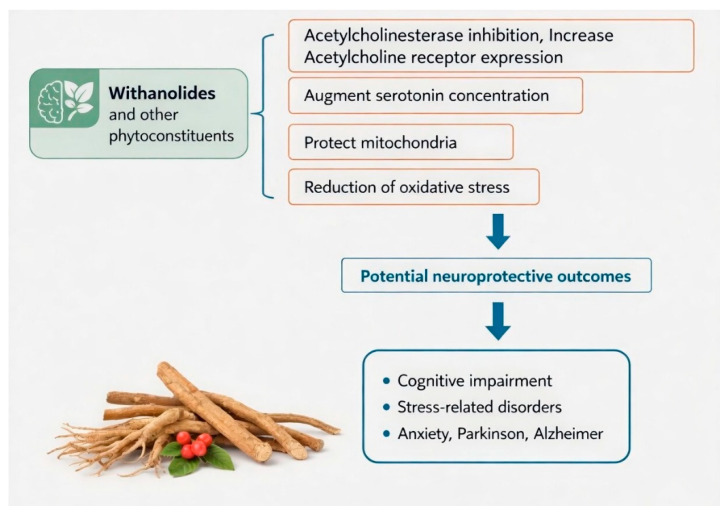
Schematic overview of proposed neuroprotective mechanisms and associated CNS-related outcomes of withanolides and other phytocompounds in WS root extracts.

The long traditional use of WS as a tonic and adaptogenic remedy has garnered interest in recent years for its potential uses in addressing various central nervous system (CNS) disorders, resulting in the development and study of herbal medicine or food supplements focused on these targets. Zahiruddin et al. [7] reviewed the neuroactive phytoconstituents, pharmacological studies, mechanism of action, and patents related to neuroprotective effects of WS and its application in brain disorders. The authors highlighted that phytoconstituents of WS could be identified as potential active compounds, such as the sitoindosides VII–X, withaferin A, withanosides IV, withanols, withanolide A, withanolide B, anaferine, beta-sitosterol, and withanolide D as active compounds that are at least partially responsible for its pharmacological effects. These compounds are reported to be effective in several brain disorders, including anxiety, Alzheimer’s, Parkinson’s, Schizophrenia, Huntington’s disease, dyslexia, depression, autism, addiction, and bipolar disorders [7] (Figure 3). Furthermore, the authors noted that their literature survey did not reveal any toxic effects, suggesting that these plant-derived ingredients can be safely applied in herbal medicine and dietary supplements. Clinical studies showed that the plants can be used for the treatment of anxiety, insomnia and Parkinson’s disease [120].

In Parkinson’s disease, the plant helps to protect against injury, mainly by reducing oxidative stress, increasing dopamine (dopaminergic D2 receptor) with an improvement in mitochondrial function [103,104]. A meta-analysis of five randomized controlled trials involving 400 participants found that WS significantly improved overall sleep quality compared to a placebo [105]. People with insomnia experienced greater improvements, and the benefits were stronger with higher doses (≥600 mg/day) and supplementation periods (≥8 weeks) [106,107,108,109]. Ashwagandha supplementation can raise testosterone in adults by regulating the hypothalamic–pituitary–adrenal (HPA) axis and by reducing inflammation and oxidative stress [110,111].

As for many herbal remedies, human pilot and clinical studies are still needed to assess doses, safety, and efficacy due to the limitation of up-to-date published literature that in many cases lack a standardization of the WS used in the trial and to the difficulties involved in enrolling patients and establishing measurable outcomes. To date, the published literature has shown some evidence of positive effects and clinical efficacy, but further evaluations are needed. Considering the preclinical literature and clinical studies, for the CNS system, we can summarize that several results indicate a potential neuroprotective outcome for WS.

WS doses and duration of treatments can be very different because of extract preparations, bioactive compound concentration, and the different expected effects. A recent review summarized the dose ranges reported in several clinical trials and indicated that ranges from 125 to 350 mg, administered for periods from 4 to 12 weeks, can be considered. Some more specific examples include use for stress and anxiety at a dose of 300 mg twice a day for 60 days. Craving, stress, sleep, and well-being in students have been treated with 350 mg twice a day for 30 days [2]. Overweight middle-to-older age adults with high stress, fatigue, and inflammation have been treated with 200 mg twice a day for 12 weeks. Doses related to application in patients with CNS-related diseases have been also reported, with a dose of 250 mg in the morning for 7 days and 250 mg twice a day for 7 weeks for anxiety in adults; 500 mg once a day for 12 weeks in patients with schizophrenia; 250 mg for the first week followed by 500 mg for 8 weeks in patients with bipolar disorders; and 500 mg for the first week followed by 1000 mg for 12 weeks in patients with schizophrenia or schizoaffective disorder [2].

## 6. WS Safety and Toxicity

The growing global use of WS as a nutraceutical underscores the need for a careful evaluation of its safety profile. Although WS has a long history of traditional use, safety should be interpreted in relation to dose, extract standardization, duration of use, and individual susceptibility factors (e.g., comorbidities and concomitant medications) [118,119,120,121,122]. WS has a long tradition of use, especially in India and other Asian countries, and is considered a reasonably safe drug. The literature reports several toxicological studies that have examined the extracts of plant parts and, in some cases, pure compounds isolated from WS [5,117,120,121,123].

Preclinical studies generally report good tolerability in animal models at commonly tested doses; however, dose levels reported in toxicology studies (e.g., gram/kg/day ranges in rodents) are not directly translatable to human supplementation and should be interpreted within the specific study context (species, extract composition, duration, and endpoints) [117,123,124]. Clinical trials and meta-analyses indicate that WS is generally well tolerated over short-term supplementation periods, with most adverse events being mild and transient (e.g., gastrointestinal discomfort, nausea, drowsiness) [112,125]. In randomized controlled trials on stress and sleep outcomes, serious adverse events were uncommon at doses of around 600 mg/day over 8–12 weeks, although study designs and products varied [117,126]. Longer follow-up evidence remains limited and product-specific (e.g., standardized root extracts), and therefore cannot be generalized to all commercial preparations [127].

Based on the available clinical evidence, most randomized controlled trials evaluating WS extracts for stress-, sleep-, and CNS-related outcomes have employed supplementation periods ranging from 6 to 12 weeks, typically using 300–600 mg/day of standardized root extracts, within which WS appears generally well tolerated in healthy adults, with adverse events mostly mild and transient (e.g., gastrointestinal discomfort, somnolence) [3,105,106,107,108,109,112]. Data on continuous long-term use beyond 3 months remain limited and product-specific, and although observational studies and selected trials suggest tolerability of some standardized extracts for up to 6–12 months, these findings cannot be generalized due to variability in extract composition and withanolide profiles [121,127]. In the absence of robust long-term safety data, a precautionary approach is advisable, and cyclic use (e.g., 8–12 weeks of supplementation followed by short discontinuation periods) may be considered in nutraceutical practice, although this strategy is based on clinical prudence rather than direct comparative evidence. With prolonged or high-dose use, attention should be paid to potential adverse effects [69,121].

Despite an overall favorable short-term tolerability profile, cases of suspected herb-induced liver injury have been reported for some WS products, typically resolving after discontinuation [69,128]. Such events may relate to product-specific factors, including variability in withanolide profiles, high levels of certain constituents (e.g., withaferin A), contamination, or adulteration [69]. A review related to clinical studies and observed toxicities indicated that Ashwagandha was well tolerated, with only minor adverse effects reported [3,5]. Some reports suggest caution in specific conditions, especially due to the indication that large doses of Ashwagandha can be abortifacient and are considered unsafe during pregnancy [5]. Caution on WS use should be taken into consideration in subjects treated with antihypertensives, antidiabetic drugs, thyroid medications, and immunosuppressants [5].

Regulatory discussions in several countries reflect a precautionary approach for specific populations, emphasizing the importance of product quality control, clear labeling, and standardized manufacturing. Overall, the available literature supports cautious short-term use in healthy adults, while highlighting the need for standardized products and well-designed long-term safety studies. Emerging evidence suggests that WS may exhibit a shift from antioxidant to pro-oxidant activity under conditions of glutathione depletion, raising safety concerns. Consequently, regulatory authorities in several European countries, such as Denmark, the Netherlands, and Germany, have issued warnings or imposed restrictions on its use, particularly among vulnerable populations [121]. This highlights the importance of quality control and standardization in herbal formulations. Mechanistically, the toxicity of WS is not fully understood but may involve modulation of oxidative stress pathways, mitochondrial function, and neuroendocrine signaling. While many of its phytoconstituents exhibit antioxidant and cytoprotective effects, certain compounds may exert pro-oxidant or cytotoxic effects at higher concentrations [111,122]. Additionally, its interaction with the hypothalamic–pituitary–adrenal (HPA) axis and glucocorticoid receptors suggests potential endocrine effects that warrant further investigation, particularly with long-term use [117]. Limited evidence suggests that WS may influence thyroid function and hormone levels, indicating that individuals with endocrine disorders or those taking hormone-related medications should use it under medical supervision [123,124]. Furthermore, its safety during pregnancy and lactation has not been established, and its use is generally not recommended in these populations [129].

Opinion and regulatory indications regarding the use of WS vary globally (Table 3). This is due to the very old traditional uses in many oriental countries and the inclusion of the remedy in pharmacopeia or official government acts for many years, such as in India [130,131]. Modern use in food supplements or in fortified foods raised questions and doubts, as did the presence in some markets of herbal drugs or medicinal preparations with WS [130,131,132,133]. As a result, nice recent reviews have taken into consideration the legal status of the WS in various countries [134]. In the United States and the United Kingdom, Ashwagandha root preparations are generally available as food or dietary supplements under the respective regulatory frameworks (Dietary Supplement Health and Education Act (DSHEA) in the US and food supplement/traditional herbal regulations in the UK), without approval for disease treatment claims. The National Center for Complementary and Integrative Health (NCCIH) of the NIH considers Ashwagandha to have an acceptable safety profile for short-term use based on available clinical evidence, although it does not constitute formal regulatory approval.

A major challenge in the evaluation of Ashwagandha is the high variability among commercial products, including differences in plant chemotype, geographical origin, extraction methods, withanolide standardization, dosage, and manufacturing processes. These factors can significantly influence the phytochemical composition of final products, thereby affecting both efficacy and safety outcomes and limiting reproducibility across studies. Regulatory divergences are also evident internationally. For example, Denmark introduced precautionary restriction on Ashwagandha use in food supplements in 2024 based on risk assessments and reports from the Danish Technical University (DTU). Thus, regulatory approaches to WS differ across jurisdictions and may reflect precautionary interpretations of emerging safety signals, particularly in the context of product heterogeneity and limited long-term data. At the same time, several randomized trials and systematic reviews report benefits in terms of stress- and sleep-related outcomes using specific standardized preparations. These parallel developments highlight the need for harmonized quality standards, transparent reporting of extract composition, and robust long-term safety datasets to better inform regulatory decision-making [142,143]. WS is reported to have effects on female hormone levels [82], which have also been documented in clinical trials. This suggests that attention should be paid to its use under specific circumstances; however, it cannot be clearly stated to be abortifacient [144]. Thus, the literature up to now does not support the abortifacient effects of WS [144].

As a general point of view on the safety of WS, the available literature suggests that its extracts are generally safe for short-term use in healthy individuals. However, substantial variability in extract composition, plant parts used, manufacturing processes, dosage regimens, and potential alterations in phytoconstituents profiles indicate an urgent need for standardization of active constituents and phytochemical content in commercially available products. The use of WS supplements is expanding, and some doubts about the safety of both the root and the leaves have been raised, mainly due to the liver and reproductive system. Some case studies have reported liver injury linked to supplements containing a mixture of WS leaves and roots, making it difficult to determine which part of the plant, if any, is responsible for the toxicity. A WS safety dossier proposed by the Indian Ministry of AYUSH (Ayurveda, Yoga, Unani, Siddha, Homeopathy) concluded, based on an evaluation of 27 specific toxicity studies, that WS root extract is safe for human consumption at a concentration dose of at least 2000 mg/kg body weight, with no observed adverse effects [6].

There is a clear need for well-designed, long-term clinical studies to more definitively establish the safety profile of these preparations. Further research should focus on dose standardization, the identification of both active and potentially toxic constituents, and systematic evaluation of product-specific risks to ensure safe and effective use in nutraceutical and therapeutic applications.

## 7. The Putative Synergistic Impact of Psychobiotics on the Gut–Brain Axis

Growing research underscores the central role of the gut–brain axis in regulating mood, cognition, and stress responses through intertwined neuroendocrine, immune, and metabolic pathways. In this regard, some years ago, the definition of psychobiotics was adopted, namely, beneficial bacteria or compounds support such as bacteria prebiotics, which influence bacteria–brain relationships [145]. Within this framework, psychobiotics have gained attention for their capacity to modulate neuroinflammation, neurotransmitter synthesis, and hypothalamic–pituitary–adrenal (HPA) axis activity, thereby influencing mental health outcomes [146]. Recent systematic reviews and meta-analyses consistently indicate that probiotic supplementation leads to significant reductions in depression and anxiety symptoms across diverse populations [147].

These effects are particularly evident when probiotics are administered as an adjunct to antidepressant therapy [148]. Among the most rigorously investigated psychobiotic strains, *Lactobacillus helveticus* R0052 and *Bifidobacterium longum* R0175 have demonstrated notable anxiolytic and stress-modulating effects in both preclinical models and human studies. Evidence from human trials indicates that 30-day supplementation with this strain combination leads to improvements in somatization, depressive symptoms, and anger-hostility scores in adults [149]. Moreover, experimental findings from other studies demonstrate enhanced GABA production and anti-inflammatory cytokine responses, further supporting their mechanistic relevance to gut–brain axis communication [150]. Further studies in adults with mild anxiety demonstrate beneficial psychological outcomes and gut-microbiota changes, indicative of psychobiotic activity. Ongoing clinical trials (e.g., NCT06391216) continue to investigate the CNS-related actions of *Lactobacillus helveticus* R0052 and *Bifidobacterium longum* R0175, reinforcing their position as leading next-generation psychobiotics [151]. It is useful to note that the use of WS has been studied in the area of CNS diseases, and in a recent randomized controlled trial, authors observed reductions in stress, anxiety, and a change in cortisol levels [152]. Systematic reviews confirm WS safety and efficacy in individuals experiencing stress-related or mental health symptoms [153].

Although no clinical trials have directly evaluated combined supplementation with psychobiotics and WS, their complementary mechanisms provide a hypothesis of biological plausibility for potential synergy within the gut–brain axis; this remains to be tested in well-designed human trials [154]. Few papers have been published thus far on the specific topic of probiotics and WS. Yu et al. [155] adopted an in vitro approach considering single- or mixed-strain fermentation using four lactobacillus strains on the extract of WS. Both lactobacillus parameters, as well as the chemical composition of the extract, were studied. The results revealed that the fermentation of *L. plantarum* DY-1, *L. casei* KDB-LC, *L. acidophilus* KDB-03, and *L. fermentum* KDB-08 with WS increased the antioxidant activity and flavor components of the extract. The composition of the extract was changed, indicating that fermentation with lactic bacteria can be used as an attractive and innovative way to improve flavor and bioactivity of the WS extracts [155]. Preclinical evidence supports this hypothesis, while underscoring the need for well-designed human trials to confirm the combined efficacy.

## 8. Use in Nutraceuticals and Food Supplements

The WS market has gained in popularity, with an increase in demand, especially in the area of nutraceuticals. WS can be included in dietary supplements and functional foods enriched with bioactive compounds to enhance both nutritional and health-promoting benefits. For this reason, WS, which is traditionally known for its adaptogenic and medicinal properties, is highly regarded and its use in different products is increasing [4,9]. The introduction of WS into supplements and food products is aimed at exploiting its health-promoting properties, thereby allowing exposure of people to the plant’s bioactive compounds through products consumed as part of their diet. A specific review by Singirala et al. [9] has recently been published in this regard. At present, WS is present in multiple types of products distributed and sold in most world markets, mostly as nutraceutical or dietary supplements. Further applications will be probably developed in the near future, with inclusions in “functional foods”; stricter requirements related to the extraction process and standardization will also likely be necessary to ensure the quality and safety of such products.

## 9. Conclusions

Ashwagandha (WS) has a long history of use in many countries and has been extensively studied both from phytochemical and pharmacological perspectives. Its diverse phytoconstituents, including steroidal withanolides (such as withaferin A and withanolide A), flavonoids, and alkaloids, are largely responsible for its wide-ranging biological effects. Standardized extracts are crucial in nutraceutical formulations to ensure an adequate level of these bioactive compounds. Research has reported signals of benefit for several CNS-related outcomes (e.g., stress- and sleep-related measures), with stronger support in preclinical studies and variable clinical evidence depending on indication, extract standardization, dose, and study design. Mechanistically, WS exerts its effects by modulating the HPA axis, reducing cortisol levels, enhancing antioxidant defenses, improving sleep quality, regulating neurotransmitters and hormones such as dopamine and testosterone, and protecting neurons from oxidative and inflammatory damage. The growing number of patents on WS formulations highlights its potential therapeutic usefulness and the increasing commercial interest in herbal medicine. Furthermore, its inclusion in food supplements, functional foods, and nutraceutical products underscores its increasing relevance in promoting general health, cognitive function, stress resilience, and overall well-being. Collectively, the literature suggests that WS acts as a multi-target botanical with potential applications in health-promoting products; this warrants confirmation in standardized, adequately powered clinical studies. Future research should aim to further clarify the specific molecular pathways through which WS exerts its neuroprotective, endocrine, and adaptogenic effects. Although current findings are promising, large-scale, long-term clinical trials with standardized extract profiles are needed to better define optimal dosages, treatment duration, and safety across diverse populations. Additionally, comparative studies between different extraction methods, phytochemical compositions, and formulation technologies would help identify the most effective and bioavailable preparations.

## Figures and Tables

**Table 1:** Major phytochemical contents of the *Withania* plant parts.

Plants	Phytochemicals	Activity or Aim of the Evaluation	Plant Parts and General Amount	Data Obtained	Reference
*Withania somnifera*	Flavonoid, withaferin A, withanolide C, 4-deoxyphysalolactone,	Antioxidant, Antibacterial	Leaves flavonoids 5 mg/g Withanolides 2.0 mg/g	In vitro test on *Staphylococcus aureus* and *Escherichia coli*	[46,47,48]
	Alkaloids, withanine, withasomnine, anaferine, and tropine; withaferin A, withanolide A, withanoside IV, withanolide C, withanolide Z, withanone	Anticancer, Infertility treatment, Anti-inflammatory/immunomodulatory, Antidiabetic, Cardioprotective,	Roots Alkaloids 1–3 mg/g Withanolides 1.0 mg/g	In vitro assays, analytical analysis and ex vivo tissue animal model	[47,49,50,51]
	withasomnilide, withasomniferanolide, somniferanolide, somniferawithanolide, somniwithanolide	Isolation and structural elucidation of coompounds	Stem bark quantitative data not available	Isolation and structural elucidation of constituents	[52]
	withanamide A, withanamide B, withanamide C, withanamide D, withanamide E, withanamide F, withanamide G, withanamide H, withanamide I	Chemical characterization of plant materials, bioassays on antioxidant activity	Fruits withanolide 0.1 mg/g	Chemical profiling by NMR and GC-MS; in vitro antioxidant assay	[31,53]

**Table 2:** Summary of clinical and preclinical evidence on the effects, mechanisms, and dosage of WS.

Reference/Study Type	Study Design	Duration	Extract Type/Route/Daily Dose	Form of Extract	Administration	Main Outcome
[108]	Randomized, double-blind, placebo-controlled	10 weeks (week 5, 10)	Root extract in capsule (KSM-66)/Oral/600 mg	Capsules	Oral	Improved sleep efficiency and mental alertness
[109]	Randomized, double-blind, placebo-controlled	8 weeks (week 1, 4, 8)	Root extract in capsule (KSM-66)/Oral/600 mg	Capsules	Oral	Improved sleep efficiency and mental alertness
[106]	Randomized, double-blind, placebo-controlled	12 weeks (week 4, 8, 12)	Root extract in capsule (KSM-66)/Oral/600 mg	Capsules	Oral	Improved sleep quality and mental alertness
[117]	Randomized, double-blind, placebo-controlled	60 days (day 15, 30, 45)	Aqueous full-spectrum root extract (KSM-66/oral/300 mg)	Capsules	Oral	Stress and anxiety outcomes improved, with no serious adverse events reported
[91]	Randomized, double-blind, placebo-controlled	90 days	Root extract in capsule (Oral/300 mg)	Capsules	Oral	Reductions in stress-related outcomes and improvement in sleep quality
[118]	Randomized, double-blind, placebo-controlled	90 days (day 30, 60, 90)	Root extract in capsule (Oral/500 mg)	Capsules	Oral	Improved symptoms of anxiety and depression
[114]	Randomized, double-blinded, placebo-controlled	6 weeks (1 g/day)	Root extract in capsule	Capsules	Oral	Safe and effective adjunctive therapy for generalized anxiety disorder
[119]	Randomized, double-blind, placebo-controlled	6 weeks (week 6)	Root and leave extract in capsule (Shoden1)/Oral/120 mg	Capsules	Oral	Improved sleep quality by significantly improving non-restorative sleep (NRS) condition

**Table 3:** Global regulatory status and legal framework of WS.

Region	Legal/Regulatory Status	Classification	Key Restrictions/Requirements	Key References
India	Legal (widely used)	Ayurvedic drug + nutraceutical	Root only allowed in food supplements; licensed manufacturing under AYUSH/FSSAI	Drugs and Cosmetics Act, 1940; FSSAI Food Safety and Standards (Ayurveda Aahara) Regulations, 2022; Ministry of AYUSH guidelines [130,131]
USA	Legal	Dietary supplement	No disease treatment claims allowed; DSHEA compliance required	Drugs and Cosmetics Act, 1940; FSSAI Food Safety and Standards (Ayurveda Aahara) Regulations, 2022; Ministry of AYUSH guidelines [132,133]
European Union	Legal but restricted	Food supplement (not medicinal)	No approved Science, Safe Food, Sustainability (EFSA) health claims; some member state restrictions	The directive in EU: EU Food Supplements Directive 2002/46/EC; EFSA Health Claims Register; EU Novel Food Regulation (EU) 2015/2283 Recent paper summarizing the actual situation in the EU [134]
United Kingdom	Legal	Food supplement/traditional herbal product	Medicinal claims require Medicines and Healthcare product Regulatory Agency (MHRA) authorization	Some general information is reported in papers related to use of WS in the UK [135,136]
Canada	Legal (licensed)	Natural Health Product (NHP)	Product license Natural Product Number (NPN) required; evidence for safety/efficacy	Specific regulation based on the product registration. All summarized in Brendler et al. [121]
Australia	Legal	Complementary medicine	Must be listed/registered with TGA; strict quality control	Commonwealth of Australia Therapeutic Goods Act 1989; 1989; [137] Brendler et al. 2025 [121]
Japan	Legal (limited use)	Food supplement/traditional ingredient	Limited therapeutic claims allowed	Ministry of Justice, J. Food Sanitation Act (Act No. 233 of 1947); 2025 [138]
China	Limited/traditional use context	Traditional herbal medicine (indirect use)	Used in Traditional Chinese Medicine (TCM) frameworks but not standardized as a WS monograph drug	Pharmacopoeia Commission of the People’s Republic of china Pharmacopoeia of the People’s Republic of China (Vol. I: Traditional Chinese Medicine); Beijing, China, 2020 [139]
Nepal	Legal	Ayurvedic/herbal medicine	Less strict enforcement; traditional medicine uses common	Government of Nepal National Ayurvedik Health Policy, 2052; 1996 [140,141]

## Data Availability

No new data were created or analyzed in this study. Data sharing is not applicable to this article.
